# Successful Transhiatal Repair of Spontaneous Esophagogastric Junction Perforation Contained Within a Hiatal Hernia Sac: A Case Report

**DOI:** 10.1111/ases.70188

**Published:** 2025-11-16

**Authors:** Eiichiro Nakao, Yasunori Kurahashi, Motoki Murakami, Shugo Kohno, Yudai Hojo, Tatsuro Nakamura, Yoshinori Ishida, Hisashi Shinohara

**Affiliations:** ^1^ Department of Gastroenterological Surgery Hyogo Medical University Hyogo Japan

**Keywords:** esophageal perforation, hiatal hernia, minimally invasive surgery

## Abstract

An 86‐year‐old woman presented with repeated vomiting and hematemesis. Imaging revealed extensive mediastinal food debris accumulation, diagnosing spontaneous esophageal perforation. CT showed debris predominantly in the anterior‐right mediastinum around the esophagogastric junction, with minimal bilateral pleural effusion. Upper gastrointestinal contrast study demonstrated luminal contrast extravasation without thoracic or abdominal extension. Additional history from family members revealed that the patient had been previously diagnosed with a hiatal hernia by a physician. Given stable conditions, we hypothesized that the perforation was contained within the hernia sac and selected a transhiatal approach. Intraoperatively, an approximately 5‐cm longitudinal tear across the esophagogastric junction was identified and successfully repaired with drainage utilizing the hiatal hernia space. Unlike typical esophageal perforation progressing to severe left‐sided empyema, this case's hiatal hernia created lax esophageal adventitia, distributing pressure into the hernia sac and preventing thoracic or abdominal perforation.

## 1 Introduction

Spontaneous esophageal perforation, also known as Boerhaave syndrome, is a critical thoracoabdominal condition that requires emergency surgery with a mortality rate of at least 20% [1]. It is important to determine the optimal approach based on the location of the perforation and the extent of contamination spread, with consideration of anatomical irregularities. We present a case of spontaneous esophagogastric junction (EGJ) perforation on the anterior‐right side, with concurrent sliding hiatal hernia (HH) extending into the mediastinum that was successfully addressed via a laparoscopic transhiatal approach.

## 2 Case Presentation

An 86‐year‐old woman presented with sudden onset of repeated vomiting and hematemesis after lunch. Her comorbidities included hypertension and dementia, with histories of cerebral hemorrhage and sigmoid colon cancer surgery. She was emergently transported to a local hospital, where CT demonstrated spontaneous esophageal perforation, prompting transfer to our institution. Upon arrival, she reported mild epigastric pain without muscular guarding. Despite dementia, her consciousness was clear with stable vital signs: heart rate 62 bpm, blood pressure 124/60 mmHg, oxygen saturation 98% on room air, and temperature 36.0°C. Laboratory findings showed white blood cell count 3.8 × 10^3^/μL, C‐reactive protein 0.02 mg/dL, hemoglobin 11.9 g/dL, and D‐dimer 2.3 μg/mL.

**FIGURE 1:**
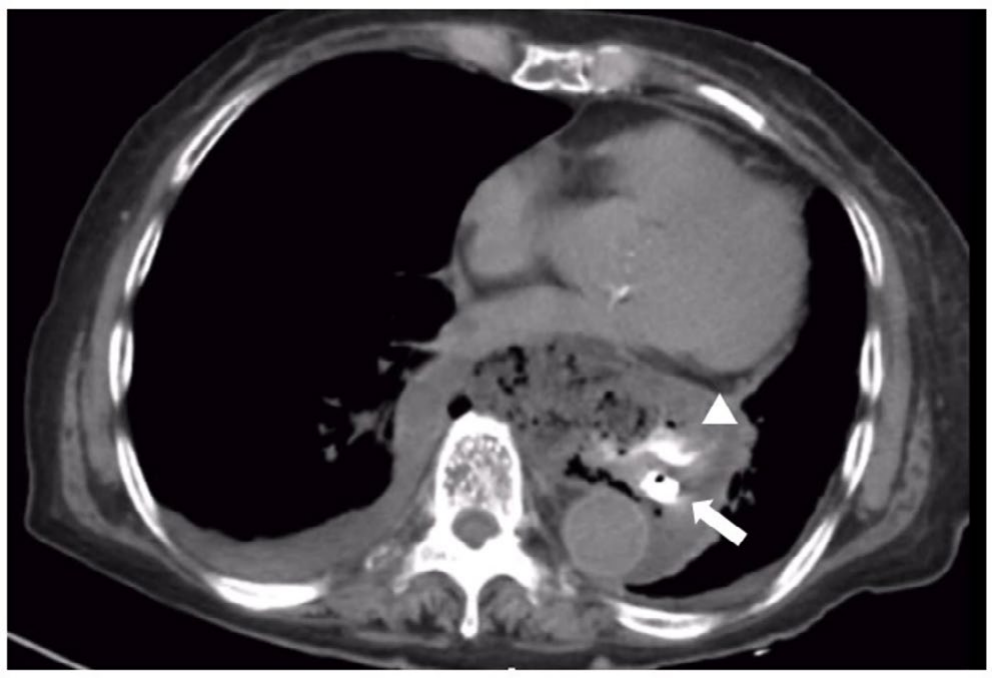
Preoperative computed tomography after upper gastrointestinal contrast study. Contrast extravasation was observed from the anterior‐right mediastinum of the lower esophagus, suggesting lower esophageal perforation (white arrowhead: Contrast extravasation; white arrow: Intraluminal contrast and nasogastric tube).

Contrast‐enhanced CT was repeated, revealing abundant food debris localized to the mediastinum. Upper gastrointestinal contrast study through a nasogastric tube showed satisfactory passage without evident extraesophageal leakage. Subsequent non‐contrast CT demonstrated contrast extravasation from the lower esophagus to the anterior‐right mediastinum, confirming lower esophageal perforation (Figure 1). Given the atypical anterior‐right location rather than typical left‐sided perforation, stable condition, and anticipated presence of HH based on the patient's history obtained from the family, an emergency laparoscopic transhiatal approach was selected.

**FIGURE 2:**
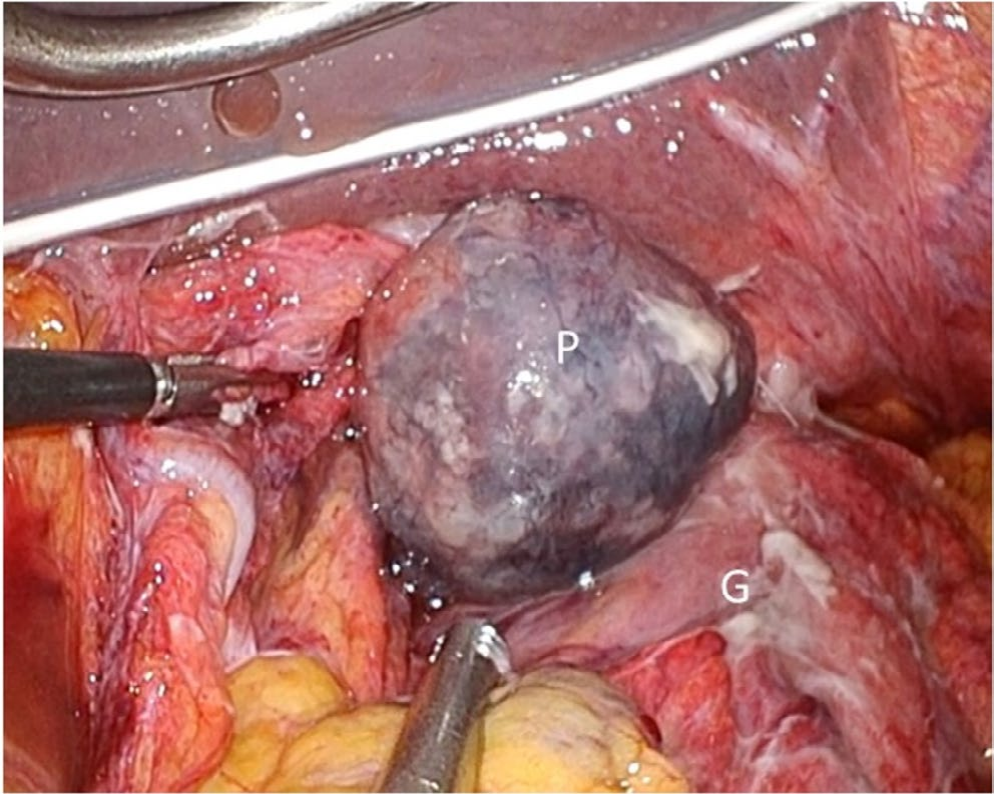
Intraoperative findings before treatment. A tense bulging of food debris covered by the peritoneum protruding into the abdominal cavity at the hiatal region was observed. No perforation into the abdominal cavity or extension of contamination was noted (P, peritoneum; S, stomach).

**FIGURE 3:**
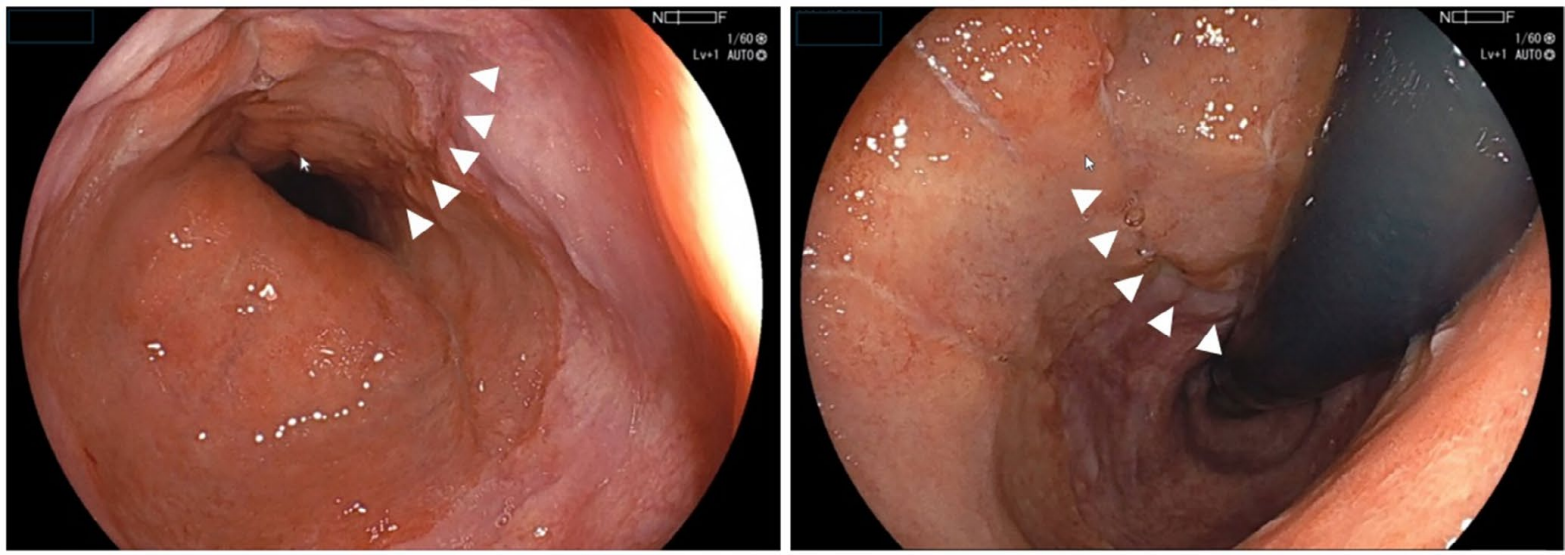
Upper gastrointestinal endoscopic findings on postoperative Day 30. The sutured perforation site shows scarring extending across the esophagogastric junction (white arrow heads, healing scar).

Laparoscopic surgery was performed using five trocars. An edited surgical video is available (Supporting Information Video S1). At the esophageal hiatus, a tense bulge of food debris covered by peritoneum protruded into the abdominal cavity without perforation or contamination spread (Figure 2). Widening of ventral and dorsal muscular bundles confirmed sliding hernia. Upon opening the mediastinal sac, food debris extending to the carina level was encountered. An approximately 5‐cm longitudinal mucosal tear spanning the EGJ on the anterior gastric wall confirmed EGJ perforation. Two‐layer suturing was performed using 3–0 monocryl. Intraoperative esophagogastroduodenoscopy (EGD) was performed, which confirmed negative air leakage testing. Food debris was thoroughly drained, and drains were placed in the mediastinum and bilateral subphrenic spaces. Operative time was 303 min. Upper gastrointestinal contrast study on postoperative Day 7 confirmed no leakage, oral intake commenced on Day 10, and the mediastinal drain was removed. EGD on Day 30 demonstrated scarring at the perforation site (Figure 3), and the patient was transferred for rehabilitation on Day 41.

## 3 Discussion

In this case, selecting the surgical approach for EGJ perforation associated with HH presented considerable challenges. However, combining preoperative imaging findings enabled prediction of the perforation site and absence of intrathoracic rupture. By selecting a minimally invasive surgical (MIS) transhiatal approach, we saved the life of an older patient without severe infectious complications.

The left lateral aspect of the lower thoracic esophagus is the most common perforation site in spontaneous esophageal perforation (Boerhaave syndrome) [2]. In the present case, contrast extravasation was on the anterior‐right side rather than the left side, allowing preoperative prediction that this case differed from typical spontaneous esophageal perforation. However, the perforation location may vary with the hernia configuration and does not necessarily indicate EGJ involvement. Although the precise perforation site was identified intraoperatively as the EGJ, distinguishing between esophageal and EGJ perforation has minimal clinical relevance regarding pathophysiology or treatment strategy. Evaluating the perforation site and rupture pattern preoperatively and responding accordingly to each case is important. The cause of perforation in this case may have been Mallory‐Weiss syndrome from repeated vomiting that progressed to EGJ perforation. Perforation patterns are classified into the intrathoracic perforation type—where the mediastinal pleura is damaged and communicates with the thoracic cavity—and the mediastinal localized type—where the mediastinal pleura remains intact [3]. The present case was the latter type. Based on stable preoperative vital signs and history of HH, we considered the MIS transhiatal approach optimal. MIS for esophageal surgery results in significant reduction in postoperative pulmonary complications, and improved outcomes are possible even for spontaneous esophageal perforation [4]. The Pittsburgh Perforation Severity Score [5] in this case was 4 points, indicating intermediate risk; however, MIS may be effective for older patients in whom blood pressure can be maintained.

**FIGURE 4:**
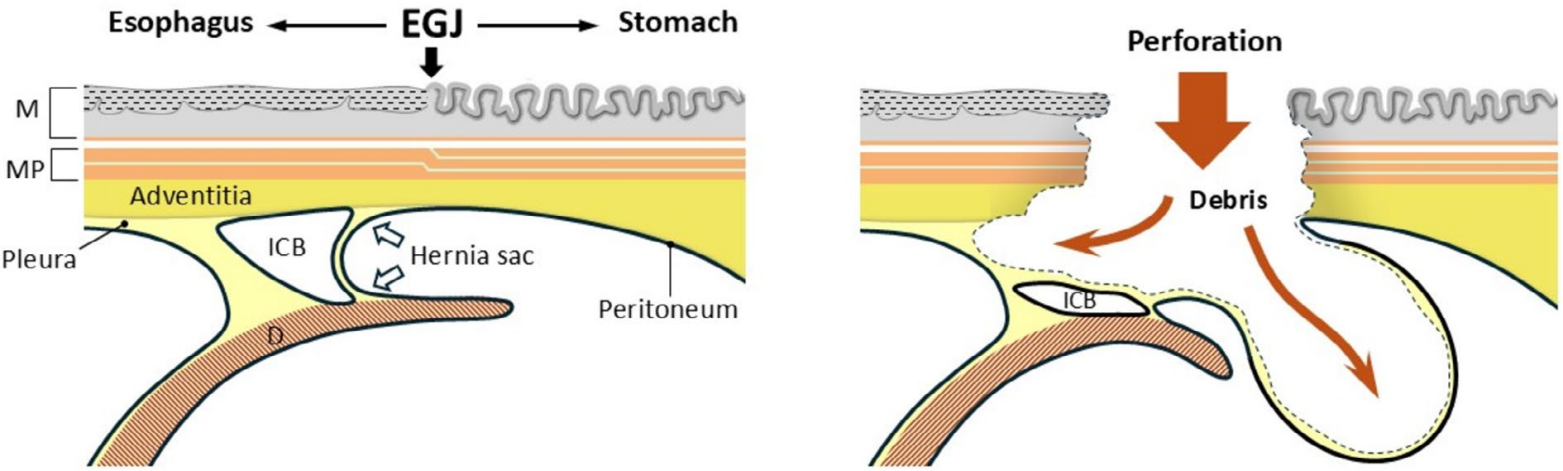
Schematic illustration of the anatomical mechanism. Food debris that perforated through the muscular layer extended by expanding the connective tissue layer of the esophageal adventitia and the hernia space created by the sliding hernia. The pressure was dispersed by expanding the mediastinal side with areolar supporting tissue and the hernia space from behind the peritoneum.

We previously reported that the esophageal adventitia is anatomically continuous with the gastric subserosa [6]. Therefore, food debris that perforated through the muscular layer was extended by expanding the connective tissue layer of the esophageal adventitia and the hernia space created by the sliding hernia, and pressure was dispersed by expanding the mediastinal side with areolar supporting tissue (Figure 4). Thus, in perforations from the esophagus to the EGJ complicated by HH, pressure is more likely directed toward the hernia space with less supporting tissue, and contaminated material may remain localized within the mediastinum without perforating the peritoneum or pleura.

Conventional esophageal perforation is relatively rare, with cases complicated by HH being excessively rare [7, 8, 9]. A PubMed search with keywords “esophagogastric junction perforation” and “hiatal hernia” for studies published from 1952 to June 2025 yielded no reported cases. There are reports of increasing trends in older patients requiring surgical treatment for HH [10], suggesting that opportunities to encounter gastrointestinal perforation within HH sacs will increase. In such cases, the transhiatal approach represents an effective approach.

In this case, we prioritized patient survival and did not perform hiatal closure or fundoplication for the HH. We were concerned that hiatal closure might result in poor mediastinitis control. Although adequate infection control might have been possible even with hiatal closure, whether hiatal closure alone can reduce the risk of recurrent herniation or gastroesophageal reflux remains debatable. Additionally, fundoplication was technically difficult due to contamination and gastric wall edema‐related thickening. Furthermore, after operations for generalized peritonitis or upper abdominal surgery, severe adhesions often develop around the hiatal region, which may reduce the likelihood of herniation of other abdominal organs.

When EGJ perforation with vomiting is complicated by HH, contaminated tissues and food debris may easily extend into the connective tissue layer of the esophageal adventitia and hernia space. The contamination may remain localized within the mediastinum without perforating the pleura or peritoneum. The transhiatal approach may be useful.

## Author Contributions

All authors meet the ICMJE criteria for authorship. Eiichiro Nakao, Yasunori Kurahashi, and Hisashi Shinohara conceived the study and wrote the manuscript. Yoshinori Ishida, Tatsuro Nakamura, Yudai Hojo, Shugo Kohno, and Motoki Murakami collected clinical data and reviewed the literature. Eiichiro Nakao, Yasunori Kurahashi, Hisashi Shinohara, and Tatsuro Nakamura performed the surgical procedure and provided critical revision. All authors read and approved the final manuscript.

## Ethics Statement

This case report was conducted in accordance with the Declaration of Helsinki. Written informed consent was obtained from the patient for publication of this case report and accompanying images. Patient anonymity was maintained throughout this study.

## Conflicts of Interest

The authors declare no conflicts of interest.

## Supporting information


**Video S1:** Laparoscopic management of esophagogastric junction perforation. This video illustrates the key surgical steps, including identification of mediastinal food debris contamination, discovery of an approximately 5‐cm longitudinal perforation at the esophagogastric junction, and successful two‐layer repair with air leak testing and appropriate drainage placement.

## Data Availability

Data supporting the findings of this study are available in the article and Supporting Information Video S1.
